# eHealth Literacy Interventions: Scoping Review

**DOI:** 10.2196/69640

**Published:** 2025-08-22

**Authors:** Yan Wang, Yutian Niu, Rongjing Xu, Qingqing Zhang, Shoumei Jia, Anni Wang

**Affiliations:** 1 School of Nursing, Fudan University, No.305 Fenglin Road, Xuhui District, Shanghai, 200032, China, 86 13467620600; 2 GuiYang Healthcare Vocational University, Guiyang, China

**Keywords:** eHealth literacy, intervention, scoping review, digital health, health literacy, eHL

## Abstract

**Background:**

Electronic resources have become a predominant modality for health information dissemination in recent years. eHealth literacy (eHL) means individuals’ competencies to effectively acquire and use health information from electronic sources. Enhancing eHL is thus essential to facilitate individuals’ effective engagement with electronic resources and promote improved health management.

**Objective:**

This scoping review aimed to synthesize the characteristics of eHL interventions, thereby providing a reference for future intervention strategies.

**Methods:**

A comprehensive search of PubMed, Embase, Cochrane Library, Web of Science, ProQuest, CINAHL, CNKI, VIP, Wan Fang Data, and Sino Med limited to Chinese and English-language studies published before August 2024 was conducted. The interventional studies included had the explicit primary objective of enhancing eHL. We also incorporated studies that assessed eHL as a secondary outcome or mediator influencing health behaviors or clinical outcomes. All publications were required to provide publicly accessible complete datasets. We excluded conference abstracts and protocols. Academic theses and dissertations were included if they underwent institutional quality assurance through rigorous academic review processes and met predefined eligibility criteria.

**Results:**

A total of 35 studies were included in this review. The most prevalent eHL interventions (12/35, 34%) were delivered via mobile apps and devices in various settings, including educational institutions, public spaces, health care facilities, and community centers. These interventions predominantly focused on enhancing information literacy, health literacy, and computer literacy across the 6 domains of eHL: traditional, health, information, scientific, media, and computer literacy. A majority of the interventions were conducted on a weekly basis (6/13, 46%) and had a duration of 24 weeks (6/35, 17%). However, 77% (27/35) of interventions did not assess long-term effects. The primary outcomes of eHL interventions encompassed perceived eHL, actual eHealth knowledge and skills, health literacy, health behavior, and clinical outcomes, with 86% (30/35) indicating positive effects. The eHealth Literacy Scale was the most frequently used assessment tool.

**Conclusions:**

This study synthesizes the characteristics of eHL interventions. Current eHL interventions exhibit limitations in theoretical grounding, longitudinal tracking, and traditional or media literacy components. Overreliance on self-reported metrics constrains validity assessment. Future work should strengthen theoretical frameworks, integrate objective metrics, and enhance longitudinal designs.

## Introduction

### Background

With the advancement of eHealth technologies, individuals increasingly access information related to diseases, treatment options, medications, and healthy lifestyles through electronic resources. As of 2024, the number of internet health care users in China has reached 365 million, constituting approximately 26% of the total population [1]. This statistic highlights the public’s reliance on eHealth resources and underscores the significant role that electronic resources play in the acquisition of health information. Nevertheless, the quality of eHealth information available is highly variable, necessitating that users possess specific skills to discern and evaluate its authenticity and reliability.

In this context, the concept of “eHealth literacy” (eHL) has emerged. Initially proposed by Norman and Skinner [2], eHL refers to the capacity to access, comprehend, evaluate, and apply health-related information from electronic resources to address health-related issues. Building upon this concept, Norman and Skinner [2] further proposed the Lily Model. According to this model, eHL encompasses 6 key domains: traditional literacy, information literacy, media literacy, health literacy, scientific literacy, and computer literacy. These 6 domains are interrelated and together form the capacity for individuals to effectively use health information in the digital environment.

eHL serves as the foundation for the effective use of eHealth resources and plays a crucial role in encouraging healthy behaviors, maintaining and promoting health, and preventing diseases [3]. However, eHL levels among populations globally require urgent improvement. A survey conducted in Northwest Ethiopia [4] revealed that over half (235/423, 56%) of the participants exhibited eHL levels below the population average. Similarly, research conducted in Germany [5] indicated that approximately 42% of surveyed individuals demonstrated inadequate performance on eHL assessments, where cancer patients particularly faced challenges in effectively navigating eHealth information. Studies from China [6,7] further substantiate this trend, consistently reporting suboptimal eHL proficiency within the general population in China. In recent years, a diverse array of eHL interventions has emerged. Conducting a review and analysis of these interventions can provide a comprehensive understanding of their implementation methodologies, application contexts, and key characteristics. This endeavor facilitates the identification of prevalent trends and existing limitations, thereby establishing a robust scientific foundation for optimizing intervention designs. Current research predominantly focuses on comprehensive analyses of individual eHL levels, measurement tools, and the relationship between eHL and health behaviors [8-10]. A preliminary search on PubMed revealed only 3 reviews on eHL interventions: 2 reviews summarized the effects of eHL interventions on older adults, both noting positive impacts on their health and management but lacking detailed intervention references [11,12], while another review summarized the application of eHL models in digital health interventions, revealing that the Lily Model has not been fully applied across its various modules [13]. Despite numerous studies investigating the effectiveness and characteristics of eHL interventions, there remains a notable absence of systematic reviews that comprehensively summarize the diverse array of these interventions. A thorough review of eHL interventions is urgently warranted to guide future research efforts and inform practical applications.

### Objectives

This study aimed to synthesize existing eHL interventions, concentrating on intervention formats, learning methods, delivery settings, components of the intervention, theory, duration, frequency, evaluation timelines of the intervention, primary outcomes and measures, and intervention effects, as well as commonly used eHL assessment tools. By identifying the characteristics and limitations of current research, this study aimed to provide a reference for health care professionals and researchers, facilitating a deeper understanding of the implementation patterns of eHL interventions and providing evidence-based guidance for future initiatives.

## Methods

### Overview

This scoping review was conducted and reported in strict accordance with the PRISMA-ScR (Preferred Reporting Items for Systematic reviews and Meta-Analyses extension for Scoping Reviews) guidelines [14]. This study did not conduct a formal critical appraisal or methodological quality assessment, as the purpose of this scoping review was to identify and map the characteristics of eHL interventions rather than to draw conclusions regarding intervention effectiveness. This checklist can be found in Checklist 1.

### Identifying the Research Question

This study seeks to address these questions: (1) What are the characteristics of eHL interventions? and (2) In which domains do eHL interventions exhibit limitations?

### Eligibility Criteria

The inclusion criteria for this review were based on the “Population-Concept-Context” framework: (1) participants of any groups; (2) for concept, the primary aim of eHL interventions was to enhance eHL itself or to improve health behaviors or clinical outcomes through its enhancement. Furthermore, this review also included studies in which eHL was assessed as a secondary outcome, allowing for a comprehensive assessment of both the interventions’ effectiveness and their impact on health outcomes; and (3) for context, potential settings for the implementation of eHL interventions encompass a range of environments, including educational institutions, residential settings, health care facilities, public libraries, and diverse digital platforms.

Furthermore, in alignment with the PRISMA-ScR guidelines [14] for scoping reviews, this study included degree theses and dissertations that were significantly pertinent to the research topic, offered detailed descriptions of interventions, and provided thorough methodological accounts, all of which had undergone institutional quality assurance through academic review processes. This inclusion aimed to mitigate publication bias and present a more comprehensive representation of the available evidence.

The exclusion criteria included (1) research proposals or conference titles; (2) studies written in non-English or non-Chinese; (3) to guarantee the integrability of the studies included and the robustness of the research outcomes, this study ruled out those with incomplete or missing raw data reports; and (4) unavailability of the full text.

### Information Sources

A comprehensive search was conducted in the following databases: PubMed, Embase, Cochrane Library, Web of Science, ProQuest, CINAHL, CNKI, VIP, Wan Fang Data, and Sino Med.

### Search Strategy

The search strategy incorporated Medical Subject Headings terms, free-text terms, and Boolean operators. Moreover, reference lists were reviewed to uncover any further relevant studies. The final search was performed on August 9, 2024. The full search strategies for each database are available in Multimedia Appendix 1. English search terms included: “digital health literacy,” “eHealth literacy,” “e-Health literacy,” “electronic health literacy,” “telehealth literacy,” “internet-based health literacy,” “online health literacy,” “health information literacy,” “health information seeking,” “health information searching,” “intervention,” “strateg*,” “program*,” “protocol,” and “practice.” Chinese search terms included: “eHealth Literacy,” “digital health literacy,” “intervention,” “program,” and “strategy.” A specific example of how the search was conducted in the PubMed database includes:

#1 was “digital health literacy” [Title/Abstract] OR “eHealth literacy” [Title/Abstract] OR “e-Health literacy” [Title/Abstract] OR “electronic health literacy” [Title/Abstract] OR “telehealth literacy” [Title/Abstract] OR “internet-based health literacy” [Title/Abstract] OR “online health literacy” [Title/Abstract] OR “health information literacy” [Title/Abstract] OR “health information seeking” [Title/Abstract] OR “health information searching” [Title/Abstract];

#2 was “intervention” [Title/Abstract] OR “strateg*” [Title/Abstract] OR “program*” [Title/Abstract] OR “protocol” [Title/Abstract] OR “practice” [Title/Abstract] OR “trial” [Title/Abstract] OR “experiment*” [Title/Abstract] OR “therapy” [Title/Abstract];

#3 was #1 AND #2.

### Selection of Study and Data Extraction

The retrieved records were imported into EndNote (version X9; Clarivate) for consolidation and duplicate removal. Two researchers (YW and YN), who were familiar with eHL interventions, independently screened the titles and abstracts based on inclusion and exclusion criteria. Next, the full texts of relevant studies were retrieved for further detailed screening. Any disagreements were resolved through discussion between the researchers, or if necessary, adjudicated by a third researcher (AW). For data extraction, the same two researchers (YW and YN) independently extracted information using a standardized form. The extracted data were then cross-checked, and disagreements were resolved through consensus discussion. In cases of uncertainty, a third researcher (AW) was consulted to make the final determination. Data were extracted on the following elements: (1) author, year of publication, country, study design, participants, sample size, and theory; (2) formats, learning methods, delivery setting and components of intervention, duration, frequency and evaluation timelines of intervention, primary outcomes and measures, intervention effects, and eHL assessment tools. A descriptive synthesis was conducted to categorize the characteristics of eHL interventions into structured tabulations (Multimedia Appendix 2 [15-49]).

## Results

### Search Results

**Figure 1.**
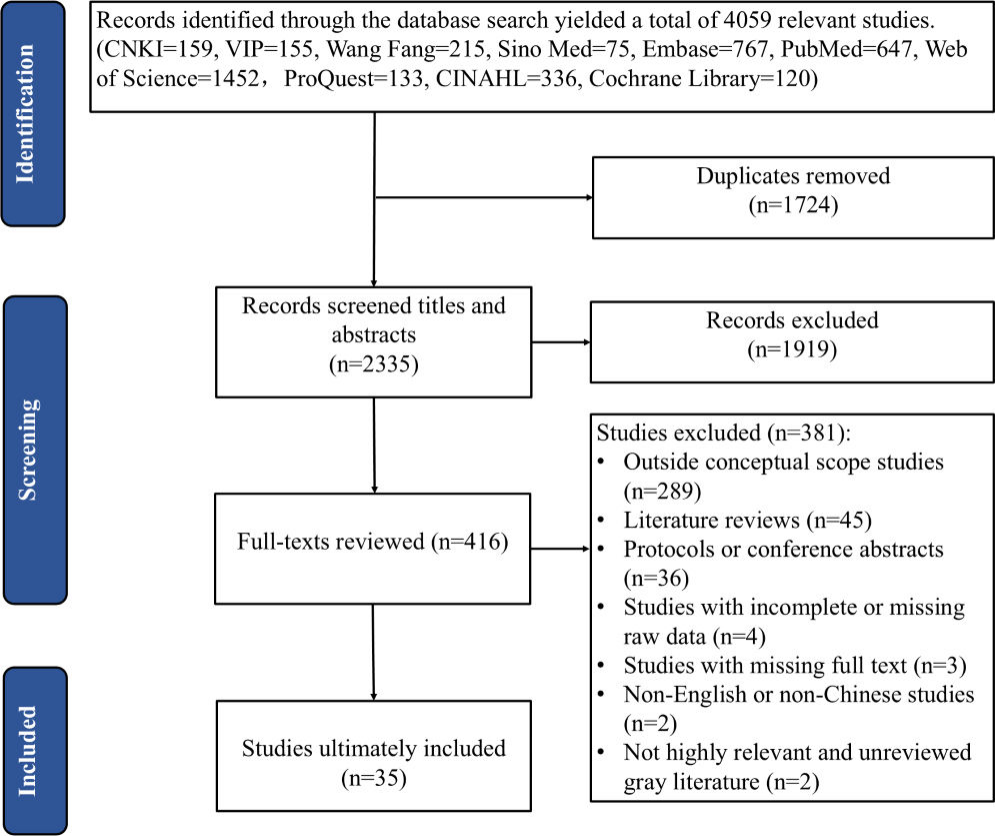
PRISMA flow diagram of the screening process. PRISMA: Preferred Reporting Items for Systematic Reviews and Meta-Analyses.

Initially, 4059 studies were retrieved. After the removal of 1724 duplicates, 1919 studies were excluded during title and abstract screening. Following a full-text review, an additional 381 studies were excluded, leading to the final inclusion of 35 studies. The detailed process is depicted in Figure 1.

### Basic Information of Included Studies

Of the 35 studies [15-49] selected, 9 studies [27-29,31,32,37-39,48] were in Chinese and 26 were in English. Of these, 9 studies [15,27-29,31,32,37,38,48] comprised master’s or doctoral dissertations, with the three most represented countries being China, the United States, and South Korea. The study designs included 12 randomized controlled trials and 23 quasi-experimental studies, with sample sizes ranging from 26 to 1389 participants. The publication years spanned from 2005 to 2024, reflecting an exponential increase in research output over this period, culminating in a peak in 2023. Participants in eHL interventions predominantly included individuals with chronic diseases, older adults, students, and those affected by infectious diseases. Among these groups, patients with chronic diseases emerged as the most frequently targeted demographic, with 16 studies specifically addressing this population. Older adults constituted the second largest group, as indicated in 8 studies. Interventions directed at students were documented in 4 studies, while 3 studies focused on individuals living with HIV. Furthermore, eHL interventions have been implemented for other distinct populations, including pregnant women, adolescents with epilepsy and their parents, adults, families, and health care professionals. Comprehensive information regarding the included studies is presented in Table 1, while specific details of the interventions are provided in Multimedia Appendix 2 [15-49].

**Table 1.** Basic information of included studies.

Author	Year of publication	Country	Study design	Participants	Sample size	Theory
Norman[Bibr R15] [15]	2005	Canada	RCT^a^	Teenagers	1389	N/A^b^
Xie [Bibr R16][16]	2011	United States	Quasi-experimental	Older people (aged ≥60 years)	124	N/A
Xie [Bibr R17][17]	2011	United States	Quasi-experimental	Older people (aged ≥60 years)	146	N/A
Xie [Bibr R18][18]	2012	United States	Quasi-experimental	Older people (aged ≥60 years)	146	N/A
Mitsuhashi [Bibr R19][19]	2018	Japan	RCT	Adults (aged 20–59 years)	282	N/A
Carroll et al [Bibr R20][20]	2019	United States	RCT	Persons living with HIV	360	Capability, Opportunity, Motivation, and Behavior model
Nokes and Reyes [Bibr R21][21]	2019	United States	Quasi-experimental	Persons living with HIV	100	N/A
Nahm et al [Bibr R22][22]	2019	United States	RCT	Older adults with chronic illnesses (aged ≥50 years)	272	Self-efficacy theory
Redfern et al [Bibr R23][23]	2020	Australia	RCT	Patients with or at risk of cardiovascular disease	891	N/A
Lotto et al [Bibr R24][24]	2020	Brazil	RCT	Parents of low socioeconomic preschoolers	208	N/A
Güvenc et al [Bibr R25][25]	2020	Turkey	RCT	Youth with epilepsy and parents	70	N/A
Hyman et al [Bibr R26][26]	2020	Canada	Quasi-experimental	Intermediate elementary students	245	N/A
Xu [Bibr R27][27]	2021	China	Quasi-experimental	Patients with systemic lupus erythematosus	41	N/A
Jiang [Bibr R28][28]	2021	China	Quasi-experimental	Patients with hypertension	60	Health as expanding consciousness theory
Guo [Bibr R29][29]	2021	China	Quasi-experimental	High-risk population for stroke	229	Ecology of Health, Self-Determination Theory
Sanders et al [Bibr R30][30]	2021	United States	RCT	Persons living with HIV	359	N/A
Zhou [Bibr R31][31]	2022	China	Quasi-experimental	Postpartum women	152	N/A
Li [Bibr R32][32]	2022	China	Quasi-experimental	Patients with nonalcoholic fatty liver disease	114	Behavior Change Wheel theory
Alt[Bibr R33] et al [33]	2022	Israel	Quasi-experimental	Undergraduate students (third year)	113	N/A
Chang et al [Bibr R34][34]	2022	South Korea	Quasi-experimental	Older people (aged ≥65 years)	46	IMB^c^ theory
Spindler et al [Bibr R35][35]	2022	Denmark	RCT	Patients with heart failure	97	N/A
Parker et al [Bibr R36][36]	2022	Australia	RCT	Patients with overweight and obesity attending primary care	215	N/A
Liu[Bibr R37] [37]	2023	China	Quasi-experimental	Women at high risk of primary osteoporosis	80	Health Promotion Model
Yang [Bibr R38][38]	2023	China	Quasi-experimental	Older hospitalized patients with coronary heart disease (aged ≥60 years)	82	IMB theory
Yu [Bibr R39][39]	2023	China	RCT	Young and middle-aged patients with stroke (aged 18–54 years)	58	Transactional Model of eHealth Literacy
Dimitri et al [Bibr R40][40]	2023	Australia	Quasi-experimental	Health care professionals	31	N/A
Son and Han [Bibr R41][41]	2023	South Korea	Cluster randomized controlled trial	Multicultural families with young children	101	ADDIE^d^ model
Roh and Won [Bibr R42][42]	2023	South Korea	Quasi-experimental	Female college students	120	N/A
Son and Kim [Bibr R43][43]	2023	South Korea	Quasi-experimental	Patients with heart failure	26	ADDIE model
Vazquez et al [Bibr R44][44]	2023	United States	Quasi-experimental	Older people (aged ≥60 years)	466	N/A
Guo et al [Bibr R45][45]	2023	China	Quasi-experimental	Patients with diabetes	132	N/A
Son et al [Bibr R46][46]	2023	South Korea	Quasi-experimental	Patients with heart failure	100	IMB theory
Kang and You [Bibr R47][47]	2023	South Korea	Quasi-experimental	Parents with children younger than 6 years of age	61	N/A
Hu [Bibr R48][48]	2024	China	Quasi-experimental	Older patients with stroke (aged ≥60 years)	76	Information ecology theory
Intarakamhang et al [Bibr R49][49]	2024	Thailand	Quasi-experimental	working-age people with risk factors for noncommunicable diseases	200	N/A

a RCT: randomized controlled trial.

b N/A: not applicable.

c IMB: Information-Motivation-Behavioral Skills.

d ADDIE: Analysis, Design, Development, Implementation, and Evaluation.

### Characteristics of eHL Interventions

#### Formats of Intervention

The most prevalent of the included studies (12/35, 34%) used digital interventions through mobile apps or devices [19,20,23,24,28,35,36,40,41,43,46,49]. Website-based digital interventions were also relatively common, comprising 26% (9/35) of the included studies [15-18,22,25,42,44,47]. In contrast, a smaller proportion of studies (8/35, 23%) used face-to-face interventions [21,26,29,30,34,37,38,45]. Additionally, fewer studies (5/35, 14%) implemented remote digital interventions using video or communication programs [27,31-33,39], along with a combination of mobile apps and face-to-face interventions [48].

#### Learning Methods of Intervention

During the intervention process, various instructional methods were used, primarily categorized into interactive learning, independent learning, and a blended approach that combines both. Interactive learning emphasizes the active engagement of learners with the learning content, tasks, or problems, encompassing communication and collaboration among learners, as well as between learners and facilitators [50]. Within the 35 studies included, a substantial proportion (24/35, 69%) used independent learning methods [18,19,21-29,31,34-36,39-43,45-48]. The blended approach, integrating independent and interactive learning, was also common, comprising 23% (8/35) of the studies [16,17,20,32,37,38,44,49]. In contrast, research concentrated on interactive learning was less prevalent, representing only 9% (3/35) of the studies [15,30,33].

### Delivery Setting

Among the 35 studies included in this review, 14 studies [16-18,20,21,25,26,30,34,36,39,44,45,48] provided comprehensive reports on the intervention settings, which can be classified into 5 primary categories: educational institutions, public learning spaces, health care facilities, welfare centers, and recreational venues. Health care facilities were the most frequently reported settings, encompassing primary care clinics [20,30], general practice clinics [36], outpatient waiting areas [25,45], and hospital wards [39,48]. A total of 7 studies [20,25,30,36,39,45,48] were conducted within this category. Public learning spaces [16-18,44] and educational institutions [21,26] were the next most commonly reported settings. Furthermore, one study was conducted at a welfare center [34] and another at a recreational venue [44].

### Components of Intervention

#### Overview

Using the 6 components of the Lily Model—information literacy, scientific literacy, health literacy, media literacy, traditional literacy, and computer literacy—the eHL intervention content of the included studies was systematically analyzed and summarized. The findings indicated that only one study [45] comprehensively addressed all 6 domains. The majority of studies (22/35, 63%) concentrated on a single domain, with health literacy being the most frequently targeted, as evidenced by 11 studies [20,23-25,28,35,36,40,46,47,49]. This was followed by information literacy and computer literacy, reported in 6 studies [15,16,21,27,32,43] and 5 studies [17,18,22,26,31], respectively.

Notably, some studies, although not encompassing all 6 domains, addressed multiple domains. For instance, one study [39] incorporated intervention content spanning 4 domains: information literacy, scientific literacy, health literacy, and computer literacy. Additionally, 3 studies [37,38,42] addressed three domains: information literacy, health literacy, and computer literacy. Furthermore, 8 studies focused on two domains, including combinations of information literacy and health literacy [19,29], health literacy and computer literacy [30,41], and information literacy and computer literacy [33,34,44,48].

#### Theory

In this study, less than half of the research (13/35, 37%) applied theories to guide the interventions [20,22,28,29,32,34,37-39,41,43,46,48]. Among these studies that applied theories, the majority of studies (7/35, 20%) drew on health behavior change fields [20,29,32,34,37,38,46], while relatively few studies incorporated theories in the fields of education and nursing training [28,29,41,43]. Additionally, apps in health informatics fields (2/35, 6%) [39,48] and health psychology fields (1/35, 3%) [22] were also limited.

The Information-Motivation-Behavioral Skills theory was the predominant framework in the domain of health behavior change, as evidenced by its application in 3 studies [34,38,46]. Additionally, the field also integrated various other theoretical frameworks, including the Behavior Change Wheel [32], the Health Promotion Model [37], the Capability, Opportunity, Motivation, and Behavior model [20], and the Ecology of Health [29]. Collectively, these theories illuminate the multidimensional aspects of behavior change, emphasizing the intricate interplay between individuals’ intrinsic motivations and their external contexts.

The theories used in the domains of education and nursing training encompass the health as expanding consciousness theory [28], Self-Determination Theory [29], and the Analysis, Design, Development, Implementation, and Evaluation model [41,43]. These frameworks underscore the significance of individual cognition and self-identity within the educational process. In the realm of health informatics, the Transactional Model of eHealth Literacy [39] and information ecology theory [48] were prominently used. Furthermore, in health psychology, the self-efficacy theory [22] was applied.

Overall, the theories underpinning eHL interventions primarily focused on individual behavior change. These theories were designed to address various aspects of individual motivation, behavior, and the surrounding environment in developing intervention strategies.

#### Duration, Frequency, and Evaluation Timelines of Intervention

The duration of eHL interventions varied significantly, ranging from 2 weeks to 1 year. Most of the selected studies (6/35, 17%) [15,24,29,32,43,46] chose a duration of 24 weeks for the intervention. This was followed by 6 weeks [26,30,42,47,49] and 12 weeks [25,28,33,39,45], with 5 studies each. In addition, a small number of studies [17-19,22,27,31,34,40,44] reported intervention durations between 2 to 5 weeks, as well as 8 weeks [37,41], and 1 year [20,23,35,36].

The majority of studies (22/35, 63%) [15,16,20-23,25,26,28,29,32,33,35,36,38-41,43,45,46,48] did not provide specific details regarding the frequency of the interventions. However, among the 13 studies [17-19,24,27,30,31,34,37,42,44,47,49] that reported intervention frequency, most interventions [30,34,37,42,47,49] were conducted once weekly, followed by those occurring twice weekly [17,18,27,44]. Additionally, one study [24] reported an intervention frequency of once every 2 weeks, while another specified a daily intervention frequency [19]. Notably, in the study by Zhou [31], the frequency of community outreach was conducted daily, whereas the intervention delivered through instructional videos occurred once per week.

The evaluation timelines for outcome indicators primarily included before and after the intervention, as well as during the intervention and follow-up. Most of the studies (18/35, 51%) [16-19,23,25,27,28,31,33,37,38,40,42,43,47-49] chose to evaluate outcomes at the two critical time points of pre- and postintervention. Additionally, 8 studies [21,22,26,30,34,41,44,45] included follow-up assessments based on evaluations conducted before and after the intervention to assess its long-term effects. Among these studies, a majority (5/8, 63%) [21,22,30,41,45] of the interventions exhibited sustained positive effects over the long term. However, 2 of these interventions [21,22] did not demonstrate statistically significant differences when compared to the control group during follow-up assessments. Furthermore, 3 additional studies [26,34,44] reported initial improvements immediately following the interventions; nonetheless, these effects did not persist throughout the follow-up periods, although the outcomes remained above baseline levels. A total of 9 studies [15,20,24,29,32,35,36,39,46] assessed outcome indicators both before and after the intervention, as well as during the intervention. Among these, only 1 study [35] reported significant positive effects in the first half of the intervention, but this effect was not evident during the remaining intervention period. Conversely, the other 8 studies [15,20,24,29,32,36,39,46] demonstrated improvements in primary outcomes compared to baseline, both during the entire intervention period and following its completion. However, in 2 of these studies [15,36], no statistically significant differences were observed between the intervention effects and the control group.

### Primary Outcomes and Measures

The primary outcomes of eHL interventions encompassed perceived eHL, actual eHealth knowledge and skills (the specific eHealth competencies being tested), health literacy, and their impacts on health behaviors and clinical outcomes. These studies used multidimensional outcomes to comprehensively evaluate the effectiveness of eHL interventions.

The majority (18/35, 51%) of interventions had only one primary outcome. Among these, the most common was perceived eHL, reported in 10 studies [15,19,21,31,33-35,39,40,48], followed by health behaviors in 3 studies [23,41,46] (the adherence to guideline-recommended medications [≥80% of days covered for blood pressure and stain medications] [23], health-promoting lifestyle [41], and self-care behaviors [46]). Additionally, 2 studies [38,47] used health literacy as the primary outcome (health literacy on chronic diseases [38] and the knowledge of medication safety [47]), 2 studies [18,22] used actual eHealth knowledge and skills (computer and web knowledge [18], and patient portal knowledge [22]), and 1 study [20] exclusively used a clinical outcome (patient activation) as the primary outcome.

Additionally, 17 interventions [16,17,24-30,32,36,37,42-45,49] involved multiple primary outcomes. Among the 11 interventions with 2 primary outcomes, 9 of these [16,17,25-28,30,42,44] included perceived eHL as one of the primary outcomes, in combination with other aspects: actual eHealth knowledge and skills [16,17,27,44], health behaviors [26,28,42], or health literacy [25,30]. Of the remaining interventions, one [24] focused solely on health behaviors and clinical outcomes, while another [49] concentrated on health literacy alongside health behaviors. Among the 4 interventions with 3 primary outcomes, 2 studies [32,36] measured perceived eHL, health literacy, and clinical outcomes, 1 study [37] assessed perceived eHL, health behaviors, and clinical outcomes, while 1 study [43] assessed perceived eHL, health literacy, and health behaviors. For the 2 interventions with 4 primary outcomes, one [45] evaluated mobile eHL, actual eHealth knowledge and skills, health behaviors, and clinical outcomes, while the other [29] examined perceived eHL, health literacy, health behaviors, and clinical outcomes.

The majority of interventions (25/35, 71%) [15,19-23,25,26,28-31,33-35,38-43,46-49], measured primary outcomes via participant self-reports, while the remaining 10 studies used either objective tests alone [18] or a combination of self-reports and objective tests [16,17,24,27,32,36,37,44,45] for outcome measures.

### Intervention Effects

Among the 22 studies [19,20,22,24,25,27-30,32,33,35,37-39,41,42,45-49] that adopted a between-group comparison approach instead of a within-participants pre-post comparison, eHL interventions significantly improved participants’ perceived eHL [19,25,27-30,32,33,35,37,39,42,45,48] (eHL [19,25,27-30,32,33,37,39,42,48], literacy in using technology to process health information [35], and mobile eHL [45]), and health literacy [25,29,30,32,38,47] (HIV-related health literacy [30], health literacy on chronic diseases [32,38], epilepsy knowledge [25], knowledge of medication safety [47], and knowledge of stroke [29]). In terms of actual eHealth knowledge and skills, the interventions notably enhanced participants’ knowledge and skills of mobile technology and the internet [45], and online health information discernment ability [27]. Regarding health behaviors, the interventions significantly promoted the participants’ health behavior [29], self-management level [28], and health-promoting lifestyle [37,41]. Additionally, they encouraged children’s dietary behavior of consuming sugar-free candy [24] and improved sufficient health behavior [49], as well as self-care behaviors [46]. For clinical outcomes, eHL interventions significantly reduced participants’ liver fat content [32], perceived stress and depression [29], controlled the severity of early childhood caries [24], and improved bone health status [37], patient activation [20], and average blood glucose level over 3 months [45].

Among the 8 studies [16-18,26,34,40,43,44] using within-participants pre-post comparisons, eHL interventions significantly enhanced participants’ perceived eHL [16,17,26,34,40,43,44]. These interventions also demonstrated significant improvements in participants’ actual eHealth knowledge and skills, including website evaluation skills [16], as well as computer and web knowledge and skills [17,18,44]. Furthermore, the interventions significantly increased participants’ knowledge of heart failure [43], while positively modifying and enhancing self-care behaviors [43].

Additionally, in 5 interventions [15,21,23,31,36], although the primary outcomes demonstrated some improvement postintervention compared to baseline, no statistically significant differences were observed between the intervention and control groups.

### eHL Assessment Tools

Of the 33 studies [15-17,19-48] that measured eHL, the majority [15-17,19-34,36-39,41-48] used the eHealth Literacy Scale (eHEALS) for measurement. Developed by Norman and Skinner [2] in 2006, the eHEALS assesses individuals’ self-perceived abilities to evaluate, locate, and apply eHealth information to address health-related issues through eight concise questions. Owing to its broad applicability and the availability of multiple language versions, the scale has emerged as the most widely used tool for assessing eHL.

In addition to the eHEALS, one investigation used the eHealth Literacy Questionnaire (eHLQ) as a measure of eHL [35]. Developed by Kayser et al [51] in 2018, the eHLQ consists of a self-administered questionnaire comprising 35 items designed to evaluate several dimensions of eHL. These dimensions include the capacity to process health information via technology, understanding of health concepts and terminology, active participation in digital services, perceived feelings of safety and control, proactive engagement with digital services, and access to effective digital resources that are tailored to meet individual needs. The validity of this instrument has been confirmed across numerous countries.

Furthermore, Dimitri et al [40] did not use any standardized instruments; rather, a custom-designed questionnaire was developed specifically to align with the objectives of the study in evaluating eHL, including the definition of eHL and the digital tools for improving the detection of growth disorders.

In summary, approximately 97% (32/33) of the studies used validated or standardized instruments for measuring eHL, thereby enhancing the reliability and validity of the research findings, as well as improving the comparability and reproducibility of the results.

## Discussion

### Principal Findings

This study synthesized and analyzed the characteristics of eHL interventions, including intervention formats, learning methods of intervention, delivery settings, components of the intervention, theoretical foundations, intervention duration, frequency, evaluation timelines of the intervention, primary outcomes and measures, intervention effects, and eHL measurement tools. The findings provided a detailed reference for the design and implementation of future intervention studies in this domain.

The formats of eHL interventions exhibited considerable diversity across various delivery settings. For instance, 2 studies [21,26] within educational environments used face-to-face intervention methods, likely due to the availability of resources and an environment conducive to in-person interactions. Conversely, 4 studies [16-18,44] conducted in public libraries used web-based interventions, leveraging the open and quiet nature of these settings, which are particularly well-suited for such approaches. In health care institutions, the predominant modalities for intervention delivery included mobile apps or devices [20,36], websites [25], and video communication programs [39]. This preference for digital interventions in health care settings may be attributed to constraints related to equipment and physical space, rendering digital solutions a more practical and scalable alternative. Among the studies included, the predominant format of eHL interventions was digital delivery via mobile apps or devices, which aligns with the findings of Bashir et al [52]. This preference can be attributed to the convenience of this approach, which facilitates intervention monitoring and enables broader scalability beyond specific study samples. Given that eHL emphasizes the capacity to locate, access, understand, evaluate, and use health information through electronic resources to address health-related issues, interventions using mobile apps or devices gain wider adoption. However, the majority of these studies [19,20,23,24,28,35,36,40,41,43,46,49] originated in high-resource countries, with merely 3 studies [24,28,49] conducted in resource-limited countries. Notably, eHL interventions in resource-limited countries predominantly adopt face-to-face offline or remote delivery via video or communication programs. This disparity underscores the persistent global digital divide: pervasive high-speed internet access and elevated smartphone penetration in high-resource countries establish an enabling environment for mobile app or device-dependent eHL interventions. Conversely, most resource-limited countries, with China constituting a notable exception, continue to face persistent challenges such as fragmented network coverage and prohibitive device costs [53]. In such settings, in-person sessions or asynchronous delivery methods could become pragmatic alternatives to mitigate access barriers. Therefore, eHL intervention designs should be context-specific, prioritizing infrastructure-appropriate implementation frameworks. Finally, although independent learning was the most commonly used intervention method in this study, a meta-analysis [54] demonstrated no significant association between the learning method and the intervention effect. This finding implies that the learning method may not be the primary determinant influencing the efficacy of the intervention.

This study, guided by the 6 domains of the Lily Model, conducted a thorough analysis of the content within eHL interventions and identified variations in the focal areas across different initiatives. Overall, these interventions predominantly emphasized health literacy, information literacy, and computer literacy. This observation aligns with Benny et al’s findings [13]. Several factors may explain this trend: first, within the fields of health care and nursing, health literacy is widely recognized as a crucial determinant of patients’ ability to manage their health and prevent diseases [55]. Many eHL interventions aim to enhance individuals’ eHL with the overarching goal of improving their overall health, which naturally prioritizes health literacy. Second, with the increasing diversification of health information sources in recent years, the significance of information literacy and computer literacy has also escalated. Notably, only one study [45] incorporated intervention components that addressed all 6 domains of the Lily Model, while traditional literacy and media literacy received the least coverage. This finding aligns with a recent review by Benny et al [13], indicating that the limited focus on these domains constrains the generalizability of the efficacy of interventions. Furthermore, this suggests that eHL research and practice demonstrate relative deficiencies in attention and investment in traditional literacy and media literacy domains. We recommend emphasizing content integrity when implementing the Lily Model to ensure intervention comprehensiveness and efficacy generalizability [13].

The theory plays a crucial role in intervention strategies, which can provide guidance for intervention design, implementation, and evaluation. Brownell [56] emphasized that an appropriate theory would help identify risk factors for specific diseases and guide the development of interventions. A systematic review by Wong and Morrison [57] revealed that a lack of theoretical support frequently resulted in inadequate systematic and targeted approaches, potentially leading to content that fails to comprehensively address the needs of the target population. However, this study demonstrated that a significant majority (22/35, 63%) of included studies failed to incorporate theoretical frameworks in their interventions, which may limit the depth of understanding and analysis concerning the mechanisms by which certain interventions may be effective or ineffective. This finding aligns with the results of Chang et al [58], which highlighted the lack of theoretical support in the formulation and implementation of eHL interventions. Notably, this finding contrasts with the results of Dong et al [12], who reported that more than half of the 7 studies included in their review used theory. This discrepancy may be attributed to the smaller number of studies analyzed in their review. Furthermore, among the 13 interventions that incorporated theories, the majority emerged from the domain of health behavior change. These theories aim to design effective interventions by elucidating individuals’ behavioral motivations, capabilities, and social environments, thereby enhancing individuals’ eHL. Therefore, future eHL interventions should place greater emphasis on the application of theoretical frameworks, enhancing the scientific rigor and effectiveness of interventions.

In terms of the duration, frequency, and evaluation timelines of eHL interventions, this study found that the most commonly used intervention duration was 24 weeks, followed by shorter durations of 6 and 12 weeks. This finding is similar to the results of Dong et al [12], who reported that interventions lasting 4 weeks or more significantly improved individuals’ eHL. The limited effectiveness of shorter interventions may be attributed to insufficient time for participants to fully absorb and apply the knowledge acquired, thereby affecting the overall impact of the intervention. In contrast, relatively longer intervention durations allow participants to repeatedly apply the learned skills in practice, thereby enhancing their confidence and capabilities, which in turn improves their eHL. In the design of intervention studies, the frequency of the intervention may significantly influence its effectiveness [59]. However, we found that most studies (22/35, 63%) omitted detailed information regarding intervention frequency, limiting understanding of the relationship between intervention effects and frequency. Among those studies that did specify frequency, the majority implemented interventions on a weekly basis. This finding provides a valuable reference for future eHL interventions. Future research should explore the specific effects of varying intervention frequencies on outcomes and assess whether the effectiveness of intervention frequencies differs across various health issues and target populations. Furthermore, the majority (27/35, 77%) of interventions did not conduct follow-up assessments of the intervention effects. This limitation may hinder researchers’ ability to fully comprehend the true effects of the interventions, consequently impacting evaluations of their long-term feasibility and effectiveness [60]. Among the studies that addressed the sustainability of intervention effects, a majority (5/8, 63%) of the interventions reported that the interventions yielded long-term positive outcomes. Notably, Nokes et al [21] and Nahm et al [22] reported no statistically significant between-group differences during follow-up, suggesting that improvements in primary outcomes may not be solely attributable to the intervention effects but could also be influenced by various other factors. Therefore, future research should continue to investigate the factors that contribute to the long-term effectiveness of eHL interventions, including various aspects such as the intervention’s form, duration, and frequency, as well as the characteristics of the target population.

The primary outcomes of eHL interventions encompass multiple dimensions, including perceived eHL, health literacy, actual eHealth knowledge and skills, health behaviors, and clinical outcomes. Notably, single primary outcomes were the most prevalent, comprising 51% (18/35) of the total, with perceived eHL being the most frequently reported at 56% (10/18). Among studies measuring multiple primary outcomes, the majority (11/17, 65%) focused on 2 types, with the combination of perceived eHL and actual eHealth knowledge and skills being the most prevalent (4/11, 36%). This finding is consistent with the results of a recent systematic review [54]. However, Watkins and Xie [11] found that most studies relied primarily on health outcomes as the main measure to evaluate eHL interventions. Compared to health outcome measures, composite indicators of perceived eHL and actual eHealth knowledge and skills can more directly and comprehensively focus on the core elements of eHL itself. This approach avoids the complexities and uncertainties that may arise from indirectly measuring eHL through health outcomes [61]. Regarding assessment tools for eHL, 3 instruments were identified: the eHEALS, the eHLQ, and custom-designed questionnaires. The findings indicated that 97% (32/33) of researchers used validated standardized self-report tools, with eHEALS being the predominant instrument. This trend aligns with the results reported by Kim et al [9]. One possible explanation for this preference is that eHEALS is more concise than the eHLQ, which contains 35 items and is available in multiple language versions. The excessive number of items in the eHLQ may lead to participant fatigue, potentially compromising the validity of the information collected. In contrast, while the custom-designed questionnaire developed by Dimitri et al [40] consists of only 10 questions, its validity has not been established, and it lacks generalizability. The results of this study showed that the majority of eHL interventions (30/35, 86%) achieved positive effects. However, notably, the assessment of primary outcome measures in most studies (25/35, 71%) relied solely on self-reported data from participants, which may compromise the reliability of findings and potentially lead to an overestimation of intervention effects [62]. Moreover, over half (23/35, 66%) were not randomized controlled trials, resulting in poor control of confounding factors. In light of this, future interventions should optimize research design and methods of outcome assessment.

### Limitations

The limitations of this review are primarily manifested in several key areas. Due to potential constraints in cross-linguistic data extraction and analytical precision, this study exclusively included English and Chinese literature. While this approach reinforces methodological rigor, future research could enhance evidence synthesis through multilingual collaboration. Nevertheless, given that most publications in this field are in English, our findings remain representative of the prevailing evidence. Furthermore, during literature screening and data extraction, this study did not formally calculate interrater reliability. While individual interpretation differences are inevitable, we implemented rigorous measures to minimize subjective bias, including predefined inclusion criteria to standardize decision-making, dual independent screening and extraction by two researchers, consensus resolution for initial discrepancies, and third-party arbitration for unresolved cases, with documented disagreement points. Our records indicate minimal divergence, as only two studies (both grey literature) necessitated arbitration during screening. Following the third-reviewer discussion, these studies were excluded from analysis.

### Conclusions

This study reviewed eHL interventions, highlighting key characteristics and limitations. Predominant frameworks used health behavior theory, with mobile apps or devices serving as the primary intervention format; a 24-week duration was most frequent. Setting selection reflected contextual adaptability: educational and health care institutions favored in-person interactions, whereas public libraries and health care systems preferred digital interventions. Challenges include inadequate theoretical guidance, a lack of long-term follow-up, and overreliance on self-reported measures. Content emphasizes health literacy, information literacy, and computer literacy, but insufficient traditional literacy and media literacy coverage may limit effect generalizability. Although most interventions reported positive outcomes, nonrandomized designs and subjective assessments weaken the strength of the evidence. We recommend that future research prioritize enhancing theoretical integration, adopting more objective assessment methodologies, and improving the long-term follow-up of intervention effects to bolster the scientific rigor and practical value of eHL interventions.

## Supplementary material

10.2196/69640 Multimedia Appendix 1 Full search strategies.

10.2196/69640 Multimedia Appendix 2 The specific details of the interventions.

10.2196/69640 Checklist 1 PRISMA-ScR (Preferred Reporting Items for Systematic Reviews and Meta-Analyses Extension for Scoping Reviews) checklist

## Abbreviations

eHEALS: eHealth Literacy Scale
eHL: eHealth literacy
eHLQ: eHealth Literacy Questionnaire
PRISMA-ScR: Preferred Reporting Items for Systematic Reviews and Meta-Analyses extension for Scoping Reviews
