# Supplementary material for: eHealth Literacy Interventions: Scoping Review
Source: Interact J Med Res. 2025 Aug 22;14:e69640. doi: 10.2196/69640 (PMC12373412; doi:10.2196/69640)
Supplement: Multimedia Appendix 1 [file ijmr-v14-e69640-s001.doc]

| **1. CNKI** |
| --- |
| (((Title/Keywords/Abstract: health literacy) OR (Title/Keywords/Abstract: digital health literacy)) AND ((Title/Keywords/Abstract: intervention) OR (Title/Keywords/Abstract: plan) OR (Abstract: strategy))) |
| **2. WANFANG DATA** |
| (((Subject: eHealth literacy) OR (Subject: digital health literacy)) AND ((Subject: intervention) OR (Subject: plan) OR (Subject: strategy))) |
| **3. VIP** |
| (Keywords: eHealth literacy OR digital health literacy) AND (Title/Keywords/Abstract: intervention OR plan OR strategy) |
| **4. Sino Med** |
| (“eHealth literacy” OR “digital health literacy”) AND (“intervention” OR “plan” OR “strategy”) |
| **5. Embase** |
| #1: ‘digital health literacy’ OR ‘eHealth literacy’ OR ‘e-Health literacy’ OR ‘electronic health literacy’ OR ‘telehealth literacy’ OR ‘internet-based health literacy’ OR ‘online health literacy’ OR ‘health information literacy’ OR ‘health information seeking’ OR ‘health information searching’  #2: ‘intervention’ OR ‘strategy’ OR ‘program’ OR ‘protocol’ OR ‘practice’ OR ‘trial’ OR ‘experimental’ OR ‘therapy’  #3: #1 AND #2 ([chinese]/lim OR [english]/lim) |
| **6. ProQuest** |
| #1: title(‘digital health literacy’ OR ‘eHealth literacy’ OR ‘e-Health literacy’ OR ‘electronic health literacy’ OR ‘telehealth literacy’ OR ‘internet-based health literacy’ OR ‘online health literacy’ OR ‘health information literacy’ OR ‘health information seeking’ OR ‘health information searching’)  #2: title(‘intervention’ OR ‘strategy’ OR ‘program’ OR ‘protocol’ OR ‘practice’ OR ‘trial’ OR ‘experimental’ OR ‘therapy’)  #3: #1 AND #2 |
| **7. CINAHL** |
| #1: TI (‘digital health literacy’ OR ‘eHealth literacy’ OR ‘e-Health literacy’ OR ‘electronic health literacy’ OR ‘telehealth literacy’ OR ‘internet-based health literacy’ OR ‘online health literacy’ OR ‘health information literacy’ OR ‘health information seeking’ OR ‘health information searching’)  #2: XB (‘intervention’ OR ‘strategy’ OR ‘program’ OR ‘protocol’ OR ‘practice’ OR ‘trial’ OR ‘experimental’ OR ‘therapy’)  #3: #1 AND #2 |
| **8. Cochrane Library** |
| #1: (“digital health iteracy”):ti,ab,kw OR (“eHealth iteracy”):ti,ab,kw OR (“e-Health iteracy”):ti,ab,kw OR (“electronic health literacy”):ti,ab,kw OR (“telehealth literacy”):ti,ab,kw  #2: (intervention):ti,ab,kw OR (trial):ti,ab,kw OR (strategy):ti,ab,kw OR (experimental):ti,ab,kw OR (therapy):ti, ab,kw  #3: #1 AND #2 |
| **9. Web of Science** |
| #1: TI=(‘digital health literacy’ OR ‘eHealth literacy’ OR ‘e-Health literacy’ OR ‘electronic health literacy’ OR ‘telehealth literacy’ OR ‘internet-based health literacy’ OR ‘online health literacy’ OR ‘health information literacy’ OR ‘health information seeking’ OR ‘health information searching’)  #2: TS=(‘intervention’ OR ‘strategy’ OR ‘program’ OR ‘protocol’ OR ‘practice’ OR ‘trial’ OR ‘experimental’ OR ‘therapy’)  #3: #1 AND #2 |
